# Scoring function to predict solubility mutagenesis

**DOI:** 10.1186/1748-7188-5-33

**Published:** 2010-10-07

**Authors:** Ye Tian, Christopher Deutsch, Bala Krishnamoorthy

**Affiliations:** 1 Department of Mathematics, Washington State University, Pullman, WA 99164, USA; 2 Department of Chemistry, Portland State University, Portland, OR 97207, USA

## Abstract

**Background:**

Mutagenesis is commonly used to engineer proteins with desirable properties not present in the wild type (WT) protein, such as increased or decreased stability, reactivity, or solubility. Experimentalists often have to choose a small subset of mutations from a large number of candidates to obtain the desired change, and computational techniques are invaluable to make the choices. While several such methods have been proposed to predict stability and reactivity mutagenesis, solubility has not received much attention.

**Results:**

We use concepts from computational geometry to define a three body scoring function that predicts the change in protein solubility due to mutations. The scoring function captures both sequence and structure information. By exploring the literature, we have assembled a substantial database of 137 single- and multiple-point solubility mutations. Our database is the largest such collection with structural information known so far. We optimize the scoring function using linear programming (LP) methods to derive its weights based on training. Starting with default values of 1, we find weights in the range [0,2] so that predictions of increase or decrease in solubility are optimized. We compare the LP method to the standard machine learning techniques of support vector machines (SVM) and the Lasso. Using statistics for leave-one-out (LOO), 10-fold, and 3-fold cross validations (CV) for training and prediction, we demonstrate that the LP method performs the best overall. For the LOOCV, the LP method has an overall accuracy of 81%.

**Availability:**

Executables of programs, tables of weights, and datasets of mutants are available from the following web page: http://www.wsu.edu/~kbala/OptSolMut.html.

## Introduction

Correlations between sequence and structure influence to a large extent how proteins fold, and also how they function. Working under this premise, most computational methods used for predicting various aspects of structure and function employ *scoring functions*, which quantify the propensities of groups of amino acids to form specific structural or functional units. Scoring functions for mutagenesis predict the effects of changing one or more amino acids (AAs) on critical properties such as stability [1-4] or activity [5], solubility [6], etc. In experimental mutagenesis, one is often faced with the challenge of having to select a small subset from a large set of candidate mutations. Computational methods are invaluable for making such choices without generating all the mutants in the lab.

Most computationally efficient scoring functions analyze protein structure at the atomic level or at the AA level. Frequencies of groups of AAs in contact have widely been used to define scoring functions for fold recognition. The default choice is two body (pairwise) contacts [7-10], but three [11,12] as well as four body contacts [13-15] have also been used to define such potential energies. It is natural to expect higher order contacts to carry more information than two body contacts. Further, higher order contacts could not typically be modeled by summing up the component pairwise contacts [12,16]. Four body contacts defined using the concept of *Delaunay tessellation *(DT) [17] of protein structures have been employed for computational mutagenesis of protein stability [3,18,19] and enzyme activity [5]. The main advantage of employing DT is that it provides a more robust definition of nearest neighbors than pairwise distance calculations. DT of protein structure has also been used as a generic computational tool to analyze various aspects of protein structure such as secondary structure assignment [20], structural classification [21,22], and analysis of small-world nature of protein contacts [23].

Even though the all-atom structure of a protein is more accurate than representing each AA by a single point, the latter approach has its advantages. Apart from being simpler, the unified residue representation can be applied even when the full-atom structure is not available. This representation is also more well-suited for predicting mutagenesis, where the all-atom structure of the resulting mutant is usually not known. With protein solubility in mind, we introduce the *degree of buriedness *for three body contacts under the framework of DT, which estimates the extent of surface exposure or buriedness of contacts without measuring the actual surface areas. Notice that an efficient method for calculating solvent accessible surface areas uses *alpha shapes *[24], which is a generalization of DT, when working on all-atom models of proteins. At the same time, such surface area calculations do not consider the sequence identity of the AAs involved. On the other hand, some previous studies that included AA identities of the contacts have used arbitrary cut-off values on the associated solvent accessible surface areas to label the contacts as exposed or not [15]. The degrees of buriedness provides an efficient middle ground for analyzing the AA composition and the buriedness of contacts in the same setting.

Compared to stability or reactivity mutagenesis, collections of experimental data for solubility mutagenesis appear scarce. This is especially the case for solubility data that includes structural information. By exploring the literature, we have assembled a structural dataset of 137 single- and multiple-point mutants along with the associated increases or decreases in the wild-type (WT) solubilities. To our knowledge, this is the largest structural database for solubility mutagenesis assembled so far. Some previous studies [6,25,26] have developed computational models to predict whether a protein will be soluble or not. In contrast, we are predicting *changes *to the solubility of the protein, i.e., whether solubility increases or decreases due to mutation(s). Henceforth in this paper, when we use the term *predicting *solubility mutagenesis, we mean the prediction of whether solubility increases or decreases.

We define a scoring function to predict solubility mutagenesis based on the frequencies of triplets of AAs that have low degrees of buriedness, i.e., are predominantly on the surface. Machine learning techniques such as artificial neural networks or logistic regression [27] are often used to *train *such scoring functions on the experimental data. For binary classification problems, support vector machines (SVM) [28] have proven to be one of the most accurate machine learning techniques. The method of least angle regression (LAR) [29] to fit predictive models using the least absolute shrinkage and selection operator, or the *Lasso *[30] has also gained increased popularity recently. For our dataset, we have a much larger number of triplet types (3895 descriptors) as compared to the number of proteins (137). Hence we develop a new training method based on linear programming (LP), which combines some features of SVM and the Lasso. This LP method allows us to impose meaningful bounds on the weights as part of the learning process. As such, we attain better performances than the standard SVM and Lasso classifiers.

## Methods

**Figure 1:**
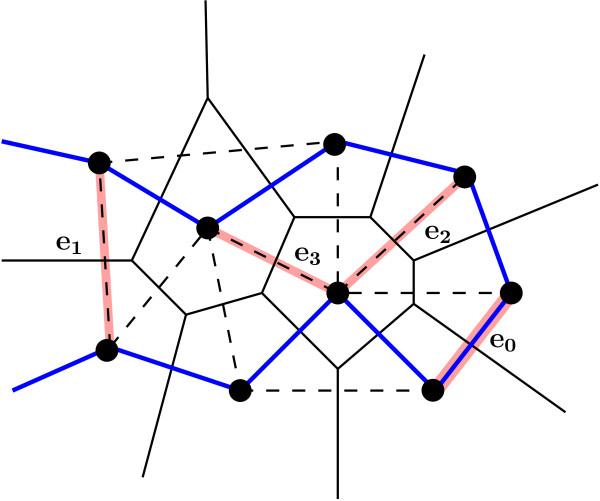
**Delaunay tessellation of a protein in 2D**. The dots represent amino acids, and the thick solid line connecting the dots is the backbone. Dotted lines are Delaunay triangles and thin solid lines represent the Voronoi cells. The four shaded edges illustrate the four degrees of buriedness for two body contacts (see Section on **Delaunay Buriedness of Contacts**). These edges are named *e_b_*, for *b *= 0, 1, 2, 3 as shown in Figure 3.

Delaunay tessellation is a construct from computational geometry that defines clusters of nearest neighbor points based on their relative proximities (see, e.g., [17]). The dual construct of DT called the *Voronoi diagram *defines convex polyhedral regions of space that are closer to the parent point than to other points. With each AA represented by a single point in 3 D space, the DT describes the structure of the protein as a collection of space-filling, non-overlapping tetrahedra (see Figure 1 for an illustration in 2D). These tetrahedra naturally define four body AA contacts. Solubility is predominantly a surface property, and surfaces are tessellated using triangles. Hence we define and analyze three body Delaunay contacts.

### Three-body Delaunay Contacts

**Figure 2:**
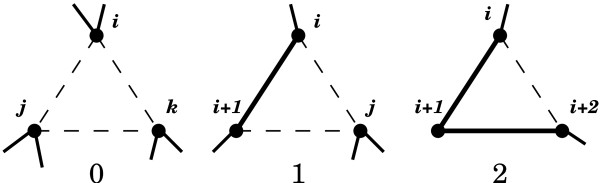
**Backbone connectivity classes for three body contacts**. *i*, *j*, *k*, etc., are residue numbers. The connectivity indices (0, 1, 2) are ordered from most non-bonded to most bonded, or connected.

Each Delaunay tetrahedron naturally defines six edges and four triangles. We define three body AA contacts using the Delaunay triangles. We differentiate the contacts based on their AA composition without considering the order in which the AAs occur in the protein sequence. This definition is motivated by the observation that contacts are often formed by AAs distant along the backbone chain, but are close to each other in 3 D space. Backbone chain connectivity is an important aspect of the contacts, though, as demonstrated by the performance of four body scoring functions [3,14]. Hence we include backbone chain connectivity as a separate factor in the definition of three body contacts. We define three connectivity classes for three body contacts, having zero, one, or two bonded edges in the triangle (see Figure 2). We appropriately index the three body connectivity classes as 0, 1, 2. Notice that for the three body connectivity class 1, the bonded edge could either be lower down or higher up along the sequence, i.e., the residue numbers could be (*i*, *i *+ 1, *j*) with *j *>*i *+ 1, or (*i*, *j*, *j *+ 1), with *j *>*i*.

### Delaunay Buriedness of Contacts

Surface exposure of AA contacts is typically determined by solvent accessible surface area calculations [15]. Since we use a unified residue representation, it is more natural to consider levels of surface exposure from a combinatorial point of view. Any two Delaunay tetrahedra from the DT are non-intersecting, or intersect at a triangle, edge, or just a vertex. Thus, each Delaunay triangle is shared by at most two tetrahedra. We define a triangle to be *Delaunay buried*, or simply *buried*, if it is part of two tetrahedra in the DT. A triangle that is part of at most one tetrahedron is hence non-buried, or is on the surface. When a triangle is non-buried, we define each of its three component edges and three vertices as non-buried. To complete the definition, we say that an edge (or a vertex) is buried if it is not non-buried. Notice that the buriedness of edges is defined using the buriedness of the three body contacts of which the edge is a component. Thus a vertex or an edge is non-buried if it is part of at least one non-buried triangle.

**Figure 3:**
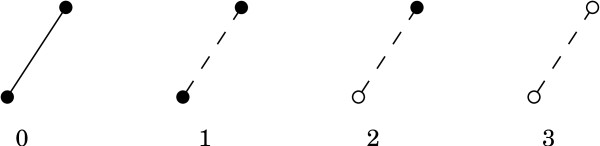
**Buriedness classes for two body contacts**. White/dotted elements are buried and black/solid elements are on the surface. Note that in Figure 1, solid lines represent the backbone of the protein.

Once we have determined whether each vertex, edge, and triangle are buried or non-buried, we can define various levels of buriedness for two and three body contacts. We first introduce the case of two body buriedness, as the buriedness of three body contacts depend on the buriedness of the component two body contacts. Further, by studying the two body case first, the reader can develop some intuition for the definitions. We define *four *levels of Delaunay buriedness for two body contacts, based on how many of the three simplices - two vertices and the edge connecting them - are buried. We appropriately index these buriedness classes by 0, 1, 2, and 3, based on the number of component simplices that are buried (see Figure 3). We also illustrate the occurrences of the two body buriedness classes in 2D in Figure 1. Interestingly, we can define the same four buriedness classes for two body contacts in three dimensions as well.

**Figure 4:**
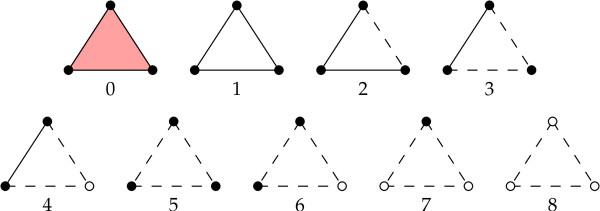
**Three body Buriedness classes**. White/dotted elements are buried and black/solid elements are on the surface. Thus the solid triangle type 0 is fully on the surface - the face, three edges, and three vertices, all are on the surface.

We now extend the definition of buriedness classes to three body contacts. This classification describes the various ways in which the vertices, edges, and the face of each triangle can be located on the surface of the protein, as described by its DT. For example, two vertices may be buried with the third one on the surface, or all three vertices and edges may be on the surface with the face buried, and so on. Altogether, there are nine buriedness classes for three body contacts (Figure 4), indexed 0-8, which range from completely non-buried (class 0) to completely buried (class 8).

**Figure 5:**
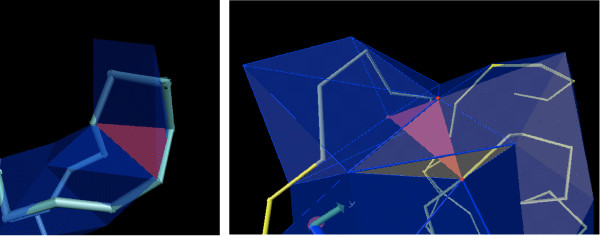
**Triplet buriedness classes 1 and 5**. Instances of triplet buriedness class 1 (left) and 5 (right), shown in red. The tube represents the backbone, and Delaunay triangles are shown in blue. The class 1 triplet is formed by the residues 7LYS, 8PRO, and 10GLN in the protein 1VQB. The class 5 triplet is formed by residues 6LEU, 53GLY, and 86ILE in the protein 2ACY. Images generated using the package VMD [43]. It is best to visualize these as well as other triplet types in 3 D. Scripts to draw all the triangles for the above two proteins in VMD are made available on the web page for the paper [37]. The reader is encouraged to load the PDB file, run the script, and then rotate the molecule appropriately in 3 D in order to visualize the same.

It is straightforward to visualize how some of the buriedness classes occur in proteins, for instance, classes 0, 4, or 8. But other classes may not be as intuitive, e.g., class 5 where the three vertices are on the surface, but the three edges and the triangle are buried. We illustrate buriedness classes 1 and 5 in Figure 5, which happen to be the two most rare classes. We do observe all nine classes in proteins.

Note that in defining the buriedness classes, it is not our goal to estimate any portions of the solvent accessible surface area (SASA) [31]. One could imagine a method that estimates the fraction of SASA that is accessible to a particular residue, and defining its buriedness based on this fraction. In comparison, our simplified definition of buriedness for a single residue is given in the framework of DT. The Voronoi tessellation, which is the direct dual of DT, has been used for accurate SASA calculations in the past [32]. At the same time, such methods work at the atomic level rather than represent each residue by a single point. The latter method of using a unified residue representation has been utilized to speed up SASA calculations [33]. The definition of buriedness classes for groups of three residues given here is combinatorial. It is different from typical SASA calculations at atomic level, and is defined specifically in the framework of DT with residues represented by single points.

#### Distance Cutoffs

The DT is originally constructed without using any distance cutoffs. Still, we need to screen the tetrahedra using a preset distance cutoff in order to define biochemically relevant AA contacts. We used a distance cut-off of 9 Angstroms for the 3-body contacts, in order to capture all the relevant surface features of the protein. We developed the entire scoring function using a dataset of sequentially diverse set of 3988 protein chains with at most 25% pairwise sequence identity at least 2Å resolution, selected by the PISCES server [34]. For this dataset, the relative frequencies of occurrence for the nine triplet buriedness classes 0-8 are 24.6%, 1.3%, 14.4%, 12.4%, 17.2%, 3.2%, 10.9%, 11.7%, and 4.3%, respectively. Thus, the surface triangles are the most frequent buriedness class. The corresponding frequencies for the three connectivity classes 0-2 were 48.2%, 43.1%, and 8.6%, respectively, showing that the non-bonded class is the most frequent one.

##### Assigning Buriedness Classes

The DT is first computed using the quickhull algorithm (using code adapted from the program of [35]). The triangles are listed by running through the list of tetrahedra (four per tetrahedron). It is a non-trivial task to fix the buriedness classes of vertices, edges, and triangles, and we need the buriedness indices of vertices and edges to fix the same for the triplets. We do all the assignments as per the definitions illustrated in Figures 3 and 4 by first creating the list of all triangles, and subsequently running through the list two more times. Hence we access the entire list of triangles *thrice*.

In fact, we maintain the faces (triangles) in two separate lists - one of buried faces and the other of surface faces. We create these lists by first running through all the tetrahedra, marking the occurrences of each face in the process. If a face is spotted for the first time, we set the buriedness class of the face as non-buried (i.e., on the surface), and add it to the list of surface faces. If we spot a face for the second time, we update its buriedness class to buried, and move this face from the list of surface faces to the list of buried faces. We then make a second run through the two lists of faces in order to assign the buriedness classes of component simplices (edges and points). Note that an edge or a vertex is non-buried if is a component of *at least *one non-buried triangle. Hence we first run through the list of buried faces and mark each subsimplex as buried. We then run through the list of surface faces, and mark each subsimplex as non-buried. The buriedness class of each vertex and edge is assigned at the end of this pass. We can now run through the lists of faces again to assign the triplet buriedness classes. We do so when we run through the list of faces for calculating the scoring function. As such, we can assign the buriedness classes for all simplices and calculate scores for them in *three *passes through the lists of all faces. Since each tetrahedron in the DT contributes at most four triangles (typically less, once we account for buried triangles), we can assign the buriedness classes of all simplices in *O*(*T*) time, where *T *is (an upper bound on) the number of tetrahedra in the DT of the protein. Notice that the space required for storing all the information pertinent to the faces is also *O*(*T*).

### Scoring Function for Solubility Mutagenesis

DT-based scoring functions have been used for predicting the effects of mutations on the stability [3,18,19], and on the reactivity of proteins [5]. Computational approaches that use structural information to predict the effects of mutagenesis on protein solubility have been rare. We hypothesize that the propensities of individual or groups of amino acids to be on the surface of a protein play vital roles in determining its solubility. With the definition of buriedness classes of triplets using the DT of proteins, we have a natural way to define scoring functions based on groups of surface residues for predicting the effects of mutagenesis on solubility of proteins.

We generalize the four body log-likelihood score defined earlier by Krishnamoorthy and Tropsha [14] to the three body case, and add buriedness classes. The score of a triangle with amino acids *i*, *j*, *k*, connectivity class *c*, and buriedness class *b *is given by

(1) $$Q_{ijk}^{cb}=\log\left[\frac{f_{ijk}^{cb}}{p_{ijk}^{cb}}\right].$$

The frequency term

$$f_{ijk}^{cb}=\frac{\text{number of }(ijk)-\text{triplets }\;\text{of classes }c\text{ and }b\text{ in dataset}}{\text{total number of type }cb\text{ triplets in dataset}}$$

represents the observed frequency of triangles in connectivity class *c *and buriedness class *b *consisting of amino acids *i*, *j*, and *k *in a dataset of proteins used to develop the scoring function. The expected frequency term

$$p_{ijk}^{cb}=Ca_{i}a_{j}a_{k}p_{cb}$$

represents the statistical expectation of encountering the triangle type, where

$$a_{i}=\frac{\text{number of amino acids of type }i\text{ in dataset}}{\text{total number of amino acids in dataset}},$$

and

$$p_{cb}=\frac{\text{number of type }cb\text{ triplets in dataset}}{\text{total number of triplets in dataset}}.$$

Note that the index *c *takes values 0, 1, 2, while the index *b *takes values from 0-8. The combinatorial factor *C *accounts for certain duplicate versions of triplets [14]. As mentioned previously under **Distance Cutoffs**, the log-likelihood ratios are estimated using a large, sequentially diverse set of proteins. This set of proteins is independent of the set of 137 solubility mutants we have assembled, which is described below.

Since we are characterizing solubility, we define the total score of a conformation as the sum of log-likelihood scores of individual triplets belonging to the five most non-buried classes of triangles, i.e., *b *classes 0-4 (see Figure 4). We define the *score *of a mutation as the total score of the mutant conformation minus the total score of the WT. We assume the WT structure (in terms of the sidechain centers of residues) for the mutant protein as well, but the identity of the mutated residues are changed accordingly. Hence, we can calculate the score of a mutation by finding the change in the total score of only the subset of triangles that see a change in amino acid composition due to the mutation. Note that single and multiple point mutations are handled in a unified way by this method. Finally, we correlate a positive (negative) score of mutation with an increase (decrease) in solubility of the protein.

### A dataset of solubility mutants

Scoring functions similar to ours are often optimized by learning from a training set of mutations [1,5,6]. At the same time, unlike the case of stability mutagenesis for which databases such as ProTherm [36] are already available, or reactivity mutagenesis for which some datasets have been assembled [5], solubility mutagenesis data with structural information has not been presented in a unified manner previously. We have assembled the largest such dataset as yet, consisting of of 137 single- and multiple-point mutants along with data on changes to their solubilities. The mutants were assembled from fifteen different studies - see Table 1 for a summary. Complete details of the dataset, including PDB codes and chain identifiers, are available in Additional File 1 (Excel), and also from the web page for the paper [37]. We identified several more studies on solubility mutagenesis (e.g., [38]), but could not include the mutants as structural information was not available for the WT.

**Table 1:** Dataset of mutations studied.

#	Article	Study	Mutants	Pred	TOT
1	[42]	Mutagenesis experiments for APOBEC3G	L260A, C261A, W168A, C281A, C288A, C308A L234A, L235A, F241A, L253A, L371A	9	11

2	[44]	AA replacement improving solubility	N159D	0	1

3	[45]	AA Contribution to solubility	Y76 D, Y76R, Y76 S, Y76E, Y76K, Y76G, Y76A, Y76 H, Y76N, Y76P, Y76C, Y76 M, Y76V, Y76L, Y76I, Y76F, Y76W	12	17

4	[46]	mutagenesis of Ab42 s'Alzheimer's peptide	F19 D, F19E, F19N, F19R, F19Q, F19 H, F19T, F19G, F19K, F19P, F19 S, F19A, F19C, F19 M, F19W, F19Y, F19L, F19V, F19I	18	19

5	[47]	Polymerization and solubility of recombination	E6F, E6W, E6L	2	3

6	[48]	Genetic selection for protein solubility	(H6Q/V12A/V24A/I32M/V36G), (V12A/I32T/L34P), (V12E/V18E/M35T/I41N), (F19S/L34P), (L34P), (F4I/S8P/V24A/L34P), I32S	6	7

7	[49]	Isolation of viral coat protein mutants	(A26T/I118F), N27 S, A107T (N24S/C46R/A96V/N116S), Q109L, (V48A/Q109H), I104V, (N12D/S34G/S52P/I92M/C101R/Q109L/S120T), (A21S/N24D/Q40R/V79A), (Q6L/N12D/I33T/R56C/F95L), (T15N/N24S/V29A/W32C/T45S/I60T/N98Y/I104N/S126P), (V61E/L103F/K106R/Y129H), (F4S/W32R/Q50R)	13	13

8	[50]	Improved solubility of TEV protease	(T17S/N68D/I77V), (T17S/R80S)	2	2

9	[51]	Primary structure and solubility	W131A, V165K, A104T, Y203 H, W140F, C19Y, P28T, V32 M, G36R, T288 M, A384P, C70 S, C26 S, C93 S, W140K, W140L, W140C, (W86F/W140F), (W130F/W140F), P28K, H44Y, (W86F/W130F/W140F), R68C, G346 S, G349 S, A198V	21	26

10	[52]	Substitutions affecting protein solubility	K97R, (K113F/W140K), (K113F/W140L), (K113F/W140C), K63 M, L104 M, T90A, L87 M, (T90A/E97A), L127 M, V74F, E97A, K69 M, (T345L/M358R), M358L, K97G, K97V, W140C, L10N, L10 D, L10T	12	21

11	[53]	Dual selection for functionally active mutants	(Y35Q/F37R), (Y35L/F37T), (Y35G/F37L), (Y35L/F37R), K27E	4	5

12	[54]	Assay for increased protein solubility	K185F, K185I, K185V, K185L, K185N, K185D	6	6

13	[55]	Phage T4 vertex protein gp24	(E89A/E90A)	1	1

14	[56]	Human cell surface receptor CD58	(Q21V/S85T/S1F/K9V/K58V/G93L)	1	1

15	[57]	Solubility and folding of a genetic marker	W232E, Y242E, I317E, (G32D/I33P)	4	4

Key: Multi-point mutants have each substitution separated by "/", and the entire mutant enclosed within braces. Pred gives the number of mutants correctly predicted by the LP-based method, out of the total number given under TOT.

We are predicting whether the solubility of the WT protein increases or decreases following a mutation. Hence we have tried to select mutants in the dataset that are soluble both before and after the mutation, but the extent of solubility changes. We have the info about whether the mutant is soluble for all except 16 out of 137 mutants in our dataset (this information was not available in the literature for these 16 mutants). From among the 121 mutants with info, only two were reported to become insoluble post mutation. Thus for most mutants in our dataset, the change in solubility reported is indeed an increase or a decrease in the WT solubility. We have also tried to find out what happens to the stability of the WT post mutation along with the change to its solubility. But this information appears often to be not reported in the literature for these mutants. We have this information for 26 of the mutants in the dataset, and among these mutants we see all four possible cases - with increase or decrease for both solubility and stability. As such, we believe that the changes in solubility and stability are independent for the mutants in our dataset.

### Training using linear programming

SVM is the standard machine learning tool used for binary classification. SVM finds a hyperplane (or a hypersurface when using nonlinear kernels) that separates the two classes of data points with maximum margin. Treating each triplet type seeing changes due to mutation as a descriptor, we have a total of 3895 descriptors for the 137 mutants in the dataset. The standard procedure for training and testing is *k*-fold cross-validation. Leave-one-out cross validation (LOOCV) is the most comprehensive, but often computationally intensive, version of cross validation (CV) using *k *= 137, i.e., with each fold containing only one protein. Two other modes popularly used for cross validation are 10-fold and 3-fold CV. Even when we use LOOCV on our dataset, there are triplet types that occur only in the single test protein, but do not feature in any of the training set mutations. We refer to such triplets as singleton triplets. SVM, or any other standard machine learning method, cannot learn the weight of a singleton triplet from the training set. Hence we propose a direct linear programming (LP) approach to do the training, in which we impose meaningful bounds on the training weights. The motivation for this step comes from the similar step in the Lasso regression [30].

For ease of notation, we index the triplets by their *type t *= (*i*, *j*, *k*, *c*, *b*), where *i*, *j*, *k *are the amino acids, and *c*, *b *are the connectivity and buriedness indices. Assuming the AA composition of triplet *t *is changed by the mutation, its contribution to the mutation score is ± *w_t_Q_t_*, where *w_t _*is the weight for the log-likelihood score *Q_t _*(Equation (1)). The sign is + if the triplet is in the mutant and - if in the WT.

Note that the default value of each type *t *is *w_t _*= 1 before training, where the contribution of each triplet is weighed equally and completely. Hence we impose the bounds 0 ≤ *w_t _*≤ 2 for each weight in our linear program. Similar to the optimization model used in SVMs, our objective function is to maximize the minimum margin, as shown in the LP below. In the training set of mutants, we denote the subset of instances seeing increase and decrease in solubility by *I *and *D*, respectively. For protein *i*, we also denote the triplet types in the mutant that see any changes by *M_i_*, and the same set for the WT by *W_i_*.

(2) $$\begin{array}{cccc} \max & \mu & & \\ \text{s}.\text{t}. & \sum_{t\in M_{i}}w_{t}Q_{t}-\sum_{t\in W_{i}}w_{t}Q_{t} & \geq & 1+\varepsilon_{i,}\;\forall i\in I;\\ & \sum_{t\in M_{i}}w_{t}Q_{t}-\sum_{t\in W_{i}}w_{t}Q_{t} & \leq & -1+\varepsilon_{i,}\;\forall i\in D;\\ & \mu & \leq & \varepsilon_{i,}\;\forall i\in I,D;\\ 0\leq & w_{t} & \leq & 2,\forall t. \end{array}$$

The variable *μ *models the minimum margin over all instances, i.e., in the optimal solution, it will be equal to the smallest *ε_i _*value. Once we get the optimal weights by solving this LP over the mutants in the training set, the score of a test protein *j *is calculated as $s_{j}=\sum_{t\in M_{j}}w_{t}Q_{t}-\sum_{t\in w_{j}}w_{t}Q_{t}$, after setting *w_t _*= 1 for any singleton triplet type *t*. The solubility of the test protein is predicted to increase if *s_j _>*0 and decrease if *s_j _*< 0.

#### Comparison to SVM and Lasso models

The standard optimization model used by SVM does not impose any bounds on the weights *w_t_*. In our LP model, the weights of triplet types that are critical to the determination of solubility are closer to 2, while the unimportant triplets get weights assigned close to zero. Since a singleton triplet does not appear in any of the training set proteins, its value will be set to zero by the LP. In comparison, SVM methods using both linear and nonlinear kernels assign nonzero values to these weights. The key modification we make is to reset the singleton weights to the default value of 1, and use the remaining weights as set by the LP when calculating *s_j _*. Equivalently, we can incorporate this change in the weights of singleton triplets by replacing each occurrence of *w_t _*in the LP (2), and subsequently in the calculation of *s_j _*, by *w_t _*+ 1. The minimum margin of separation for positive and negative data instances may not be equal in our LP, while the SVM separating hyperplane typically has the same minimum margin for both classes. If a perfect separation of all mutants in the training set into cases of increase and decrease in solubility exists, the optimal value of *μ *will be non-negative. Further, the larger the value of *μ *> 0 is, the better the separation margin is. Also, the objective function for the LP is linear, while it is quadratic for SVM even when using the linear kernel.

The idea of imposing bounds on regression coefficients has been used previously in the Lasso regression [30], but this procedure tries a range of values for these bound(s) by creating a family of models. It then chooses the best bound(s) using cross validation. In contrast, the bounds we impose are very specific to the case of the scoring function in question, and we also do not consider a sequence of bounds. We compare our LP method to the least angle regression method [29] for building Lasso models for logistic regression. Similar to the optimization model of SVMs, the objective function in the Lasso model is also non-linear.

#### Cross validation across sequentially diverse folds

As an alternative method of cross validation, we considered the division of the dataset of 137 mutants into various subsets or folds based on sequence similarity. The idea is to explore the robustness of the scoring function across sequentially diverse families of proteins. The full dataset of mutants include 19 different PDB entries, and hence we first consider *k *= 19 folds with one protein (i.e., one PDB file) per fold. As one would expect, the mutants of the same protein are classified in the same fold according to measures of sequence similarity. When leaving one fold out for the purpose of training and testing, there are many singleton triplets. Hence we are not able to assign the weights of these triplets effectively, as they do not appear in the training set of mutants. Hence we gradually increase the number of folds for the purpose of training and testing, with the folds still created based on sequence alignment scores. We employed the sequence alignment functions available as part of the Bioinformatics toolbox in MATLAB to create the folds. We consider *k *= 30, 50, and *k *= 70 folds in this analysis. These folds are made available in Additional File 3 as well as on the web page for the paper.

##### Comparison to hydrophobicity values

We have calculated the average hydrophobicity values of the mutation site residues before and after mutation according to the definitions of Varadarajan et al. [39]. The *change *in average hydrophobicity of residue *j *is calculated as $H_{\text{av}}^{\text{Mut}}(j)-H_{\text{av}}^{\text{WT}}(j)$, where *H*_av_(*j*) is calculated as an average over a window of 7 residues (Equation [2] in the original paper [39]). We want to see if changes in solubility are correlated to changes in hydrophobicity values of the mutated residues. For multipoint mutations, we average the per-residue average hydrophobicity changes over all mutation sites. Ideally, hydrophobicity values would be expected to decrease when solubility increases, as the protein attracts more water.

## Results

Previous computational studies related to our line of work have tried to predict whether the protein will be soluble or not after mutation, rather than predict the *change *in its solubility. We still mention these results briefly. Smialowski et al. [25] have summarized the accuracies of most of these methods, all of which use only sequence-based attributes. They reported an overall accuracy of 70%, while Idicula-Thomas et al. [6] reported a slightly higher accuracy of 72%, which has been the best reported accuracy so far (these authors used a different dataset of 64 mutants).

We compare the performance of our LP model to SVM and Lasso (LAR) models. Given the size of the dataset, we are able to use LOOCV, which is often computationally expensive to perform. At the same time, there is some concern that LOOCV models may cause over-fitting. Hence we compare the three models using both 10-fold CV and 3-fold CV. We used the package LibSVM [40] to build the SVM models. For creating the LAR models, we used the function cvglmnet provided as part of the LARS software [29]. This function selects the best model for logistic regression (we choose the family as *binomial*) by using 10-fold cross validation on the training set alone. Thus we use 10-fold CV as the procedure for model selection within LAR when performing LOO, 10-fold, and 3-fold CV on the overall set of mutants. The best model thus selected in each case is then used to predict the classes for the mutants in the test set.

We report the accuracy, Matthew's correlation coefficient (MCC) [41], and precisions for both classes for each model. The statistics for LOOCV are presented in Table 2, those for 10-fold CV are presented in Table 3, and those for 3-fold CV are presented in Table 4. These statistics show that the LP method outperforms SVM and Lasso classifiers based on all three CV methods. We used the default linear kernel for the SVM classifier. All nonlinear kernel options available in LibSVM performed worse than the linear kernel in this case, typically predicting all, or most, of the mutants to be in one class. The confusion matrices for LP, SVM, and Lasso prediction models are provided in Additional File 2.

**Table 2:** Statistics for LOOCV using LP, SVM, and Lasso models.

Measure	LP	SVM	Lasso
Accuracy	0.810	0.708	0.701

MCC	0.617	0.405	0.423

Precision(class *I*)	0.762	0.661	0.909

Precision(class *D*)	0.851	0.735	0.661

**Table 3:** Statistics for 10-fold CV using LP, SVM, and Lasso models.

Measure	LP	SVM	Lasso
Accuracy	0.766	0.752	0.708

MCC	0.545	0.496	0.448

Precision(class *I*)	0.719	0.705	0.952

Precision(class *D*)	0.822	0.790	0.664

**Table 4:** Statistics for 3-fold CV using LP, SVM, and Lasso models.

Measure	LP	SVM	Lasso
Accuracy	0.766	0.686	0.715

MCC	0.529	0.359	0.452

Precision(class *I*)	0.714	0.638	0.917

Precision(class *D*)	0.811	0.722	0.673

For *k*-fold cross validation across sequentially diverse folds, we report the accuracy and MCC values for *k *= 19, 30, 50, 70 in Table 5. These folds are created using sequence alignment scores, thus grouping mutants with similar sequences in the same fold. For *k *= 19, which corresponds to leaving one protein out, the performances are not great. There are many singleton triplets under this setting, for which the optimal weights cannot be assigned by learning. The performances are better when we go to *k *= 30 folds, with the LP method achieving an accuracy of 0.64 and an MCC value of 0.28. When the number of folds is increased further, the performances are expectedly better, as the number of singleton triplets go down. For *k *= 50 folds, the Lasso models outperformed the LP models, achieving an accuracy of 0.71 and an MCC value of 0.45. In summary, the scoring functions are effective as long as we can assign weights under training for a big majority of the triplet types. No obvious correlation was observed between the changes in hydrophobicity and solubility values for our dataset of mutants. 36 out of 78 mutants seeing a decrease in solubility show an increase in hydrophobicity, and 42 out of 59 mutants with increasing solubility showed a decrease in hydrophobicity. The detailed results are available in Additional File 3 (Excel) and in the web page for the paper [37].

**Table 5:** Accuracy and MCC values for *k*-fold CV using LP, SVM, and Lasso models, when the folds are created using sequence similarity scores.

	LP		SVM		Lasso	
	
*k*-fold	ACC	MCC	ACC	MCC	ACC	MCC
19	0.504	0.289	0.569	-0.056	0.569	-*

30	0.642	0.279	0.511	-0.075	0.584	0.140

50	0.650	0.289	0.409	-0.185	0.708	0.448

70	0.686	0.364	0.650	0.269	0.708	0.448

Key: *k *= 19 represents leave one protein out CV. There was no MCC value (denoted by -*) for predictions by the Lasso model in this case, as all mutants were predicted to see a decrease in solubility.

## Conclusions

This study demonstrates that the default settings available as part of standard machine learning methods may not be appropriate for all data sets. Our LP-based method could be applied to other similar datasets, in which over-fitting may be a concern due to a large number of descriptors as compared to the number of entries in the training set. At the same time, it may not be obvious what the default weight or the bounds should be for other datasets. One could also implement the flexible treatment of weights as part of the optimization framework of an SVM model.

We are trying to expand out dataset of solubility mutants by further exploration of literature. We have already found a few mutants whose solubility is reported to be "close to WT"- for example, some mutants from the study of Chen et al. [42] (which are not included in our dataset). One way to include such mutants in our study is to expand the underlying model to include a third class of mutants that see *no change *in solubility post mutation. The prediction models would then have to be developed for multiclass prediction - 3-class to be exact, into *I*, *D*, and *N *for no change. At this point, we do not have a sizable number of mutants in the *N *class, but we plan to identify enough such mutants in the near future. At the same time, it may not be obvious how the LP model can be modified easily to handle more than two classes. The default idea would be to try the one-versus-all strategy, as used in multiclass SVM [40].

For the binary classification case, we expect the LP method to be effective even on larger datasets. The total number of triplet types considered in the scoring function is 1540 × 3 × 5 = 23100 (using 20 AAs, 3 connectivity classes, and 5 buriedness classes). Even with a few thousands of mutants in the dataset, one could expect the number of triplets seeing any changes to be larger than the number of mutations themselves. Hence, one could still hope for a complete separation of the three classes when solving the LP for the entire dataset. The current dataset is diverse, but one could re-train the weights by solving the LP on a specific family of proteins, if the goal is prediction for mutants belonging to the same family. This scoring function should perform better on test proteins within the family than the default scoring function, and poorer on ones outside it. Our method handles single- and multiple-point mutants in the same manner. In fact, it may be more accurate on multiple-point mutants, as the number of triplets involved in the mutation will typically be larger.

## Competing interests

The authors declare that they have no competing interests.

## Authors' contributions

YT carried out all the calculations presented in this paper. Some code for the three body scoring function was written by BK. CD carried out the exhaustive literature search to assemble the dataset of mutants. BK supervised all work, and also did the majority of work on writing the paper. YT had some contributions to the writing as well.

All three authors have read and approved the final manuscript.

## Authors' Information

BK is an assistant professor in Mathematics, and YT is a PhD student in Mathematics working under BK. BK has done previous work on scoring functions for proteins. CD is currently a graduate student in Biochemistry, and did research under BK as an undergraduate student previously. Part of the work related to the assembly of mutant data set was done by CD when he was an undergraduate student.

## Supplementary Material

Additional file 1 **Dataset of solubility mutants**. PDB code, chain, mutation details, and information about whether solubility increased or decreased for each of 137 mutants in the dataset. Information about whether the wild type and the mutant were soluble is included. Further, info on whether stability increased, was unchanged, or decreased due to the mutations is also included when available. Predictions by the linear programming (LP) model under leave-one-out cross validation are also listed for each mutant. Format: Excel file. Click here for file

Additional file 2 **Confusion Matrices**. The confusion matrices for predictions using LP, SVM, and Lasso using leave-one-out, 10-fold, and 3-fold cross validation (9 tables). Click here for file

Additional file 3 **Cross validation across sequentially diverse folds**. Divisions of the dataset into 19, 30, 50, and 70 folds based on sequence similarity of the mutants. Analyses of predictions using LP, SVM, and Lasso methods for each division. Accuracy and MCC of predictions reported for each case. Also included is the comparison of solubility changes to changes in average hydrophobicity values. Format: Excel file. Click here for file
