# Supplementary material for: Scoring function to predict solubility mutagenesis
Source: Algorithms Mol Biol. 2010 Oct 7;5:33. doi: 10.1186/1748-7188-5-33 (PMC2958853; doi:10.1186/1748-7188-5-33)
Supplement: Additional file 2 — Confusion Matrices. The confusion matrices for predictions using LP, SVM, and Lasso using leave-one-out, 10-fold, and 3-fold cross validation (9 tables). [file 1748-7188-5-33-S2.PDF]

Additional File 2 to Scoring Function to Predict Solubility Mutagenesis – Confusion Matrices

Ye Tian[1], Christopher Deutsch[2] and Bala Krishnamoorthy*[1]

[1]Department of Mathematics, Washington State University,Pullman, WA 99164, USA
[2]Department of Chemistry, Portland State University,Portland, OR 97207, USA.

Email: Ye Tian - ytian@math.wsu.edu; Christopher Deutsch - cdeutsch@pdx.edu; Bala Krishnamoorthy*- bkrishna@math.wsu.edu;

*Corresponding author

Table 1: Confusion Matrix for LP (LOOCV)

	Experimental classification	
	Increased Solubility	Decreased Solubility
Predicted as "Increased Solubility"	48/59	15/78
Predicted as "Decreased Solubility"	11/59	63/78

Table 2: Confusion Matrix for SVM (LOOCV)

	Experimental classification	
	Increased Solubility	Decreased Solubility
Predicted as "Increased Solubility"	39/59	20/78
Predicted as "Decreased Solubility"	20/59	58/78

Table 3: Confusion Matrix for Lasso (LOOCV)

	Experimental classification	
	Increased Solubility	Decreased Solubility
Predicted as "Increased Solubility"	20/59	2/78
Predicted as "Decreased Solubility"	39/59	76/78

Table 4: Confusion Matrix for LP (10 folds CV)

	Experimental classification	
	Increased Solubility	Decreased Solubility
Predicted as "Increased Solubility"	46/59	18/78
Predicted as "Decreased Solubility"	13/59	60/78

Table 5: Confusion Matrix for SVM (10 folds CV)

	Experimental classification	
	Increased Solubility	Decreased Solubility
Predicted as "Increased Solubility"	43/59	18/78
Predicted as "Decreased Solubility"	16/59	60/78

Table 6: Confusion Matrix for Lasso (10 folds CV)

	Experimental classification	
	Increased Solubility	Decreased Solubility
Predicted as "Increased Solubility"	20/59	1/78
Predicted as "Decreased Solubility"	39/59	77/78

Table 7: Confusion Matrix for LP (3 folds CV)

	Experimental classification	
	Increased Solubility	Decreased Solubility
Predicted as ”Increased Solubility”	45/59	18/78
Predicted as ”Decreased Solubility”	14/59	60/78

Table 8: Confusion Matrix for SVM (3 folds CV)

	Experimental classification	
	Increased Solubility	Decreased Solubility
Predicted as ”Increased Solubility”	37/59	21/78
Predicted as ”Decreased Solubility”	22/59	57/78

Table 9: Confusion Matrix for Lasso (3 folds CV)

	Experimental classification	
	Increased Solubility	Decreased Solubility
Predicted as ”Increased Solubility”	22/59	2/78
Predicted as ”Decreased Solubility”	37/59	76/78
